# Effects of oil palm monoculture and agricultural land use on shredder insects in Eastern Amazonian streams

**DOI:** 10.1007/s10661-026-15076-9

**Published:** 2026-02-20

**Authors:** Ana Beatriz Oliveira Pampolha, Josinete Sampaio Monteles, Gabriel Martins Cruz, Viviane Caetano Firmino, Leandro Juen

**Affiliations:** 1 Laboratory of Ecology and Conservation (LABECO), Institute of Biological Sciences, Federal University of Pará (UFPA), Belém , Pará 66077-830 Brazil; 2 Post Graduate Program in Ecology (PPGECO), Institute of Biological Sciences, Federal University of Pará, Belém , Pará 66077-830 Brazil; 3 Post Graduate Program in Zoology, Institute of Biological Sciences, Federal University of Pará, Belém , Pará 66075–110 Brazil

**Keywords:** EPT, Land use, Stream integrity, Functional traits, Aquatic macroinvertebrates, RDA

## Abstract

Aquatic insects of the orders Ephemeroptera, Plecoptera, and Trichoptera (EPT) are vulnerable to changes in allochthonous inputs, mainly shredders, important to leaf litter decomposition and energy flow. This study evaluated the effects of land-use patterns (forest, pasture, oil palm, and mosaic) on the abundance, richness, biomass, and proportion of shredder EPT, as well as predictors of genus distribution. The study was conducted in northeast Pará, Brazil (2011–2017). One-way ANOVA and Redundancy Analysis (RDA) were used to test land-use effects and environmental influences on genus composition. Oil palm and forest streams presented greater abundances compared with the pasture streams. The forest treatment showed more shredder species than the pasture treatment. Biomass variability increased in pastures but decreased in oil palm areas, whereas forest streams did not differ from oil palm. The relative composition of shredders across land-use types followed similar patterns: the highest proportion was observed in forest streams, intermediate values in oil palm and mosaic, and the lowest in pasture. RDA explained 34% of the variation in genus composition, which was associated mainly with fine root cover and fast flow. *Phylloicus*, *Anacroneuria*, and *Triplectides* were negatively associated with fast flow. *Triplectides* responded positively to fine root cover, whereas *Nectopsyche* and *Fittkaulus* responded positively to fast flow. Anthropogenic alterations reduce shredder biomass and diversity, destabilizing aquatic communities. Forest conversion compromises the structure and function of Amazonian streams, reinforcing shredders as key indicators for long-term monitoring and conservation.

## Introduction

Streams are environments interconnected by a dendritic network, and in addition to their complexity and dynamism, their constant natural changes contribute to variability in habitats and resources (Stanford et al., 2017, Chapter 1, p. 3). This variability supports high biodiversity, accommodating organisms with diverse environmental requirements (Stanford et al., 2017, Chapter 1, p. 3; Peckarsky & Lamberti, 2017, Chapter 18, p. 379). Riparian vegetation structures the input of organic matter into streams through vertical input (leaf fall from riparian trees) and lateral input from the bank slope (indirect) (Pozo et al., 1997; Kochi et al., 2010; Rugenski et al., 2017, Chapter 28, p. 83). Therefore, alterations in the terrestrial environment, such as deforestation, can even indirectly influence the physical and chemical characteristics of aquatic ecosystems (Juen et al., 2016; Tchakonté et al., 2015; Valente-Neto et al., 2015). The loss of riparian vegetation can cause bank instability and siltation, affecting both physical structure and limnological conditions due to increased fine sediment input (Juen et al., 2016; Tchakonté et al., 2015).

In this context, the spatial distribution and energy content of allochthonous material are key factors structuring aquatic communities (Allen et al., 2024; Bacca et al., 2023; Luiza-Andrade et al., 2023). Considering that terrestrial and aquatic environments, although distinct, are directly interconnected through reciprocal resource exchange (Allen et al., 2024), the preservation of one should include efforts to conserve the other. The preservation of both aquatic and terrestrial ecosystems is a key objective of the United Nations (specifically, Sustainable Development Goals 6, 14, and 15), given their substantial economic, social, and environmental importance (UN, 2025).

Aquatic insects of the orders Ephemeroptera, Plecoptera, and Trichoptera (EPT) spend their immature stages in lotic ecosystems (Morse et al., 2019; DeWalt & Ower, 2019; Rebora et al., 2019, Chapter 7, p. 139). They play a key trophic role, primarily as consumers, linking allochthonous material input with primary productivity. Some species also function as predators, occupying higher trophic positions within the ecosystem (DeWalt & Ower, 2019; Morse et al., 2019; Thorp & Covich, 2010). Shredders are potentially more sensitive to changes in land use and land cover patterns and to reductions in allochthonous resource input, reflecting their dependence on these resources for survival (Oester et al., 2023).

The presence of shredders is strongly influenced by external factors such as forest cover, given the positive relationship between shredder biomass and increased vegetation (Houghton, 2021; Luiza-Andrade et al., 2020). Changes in land use and land cover alter the input of allochthonous material into streams (Faria et al., 2021), thereby affecting the quality, diversity, and abundance of leaf litter (Firmino et al., 2021). Considering these shredder preferences (Gonçalves et al., 2014, Chapter 6, p. 89), the conversion of forested areas to pasture or exotic monocultures, such as oil palm, is expected to substantially influence their distribution in headwater streams. Recent research has demonstrated that shredder abundance and richness are greater in undisturbed streams, and decomposition mediated by shredders is three times higher in forested streams compared with non-forested ones (Houghton, 2021; Oester et al., 2023). However, studies in the Amazon that investigate the response of shredders to deforestation, as well as the causes of such variation, are still scarce.

In light of this, this study is aimed at evaluating how shredder distribution (abundance, richness, and biomass) responds to different land-use types, to quantify their proportional representation within the community, and to identify the environmental factors that best explain these responses. We hypothesize that the conversion of forests to anthropogenic land uses—including oil palm monocultures, pastures, and mixed-use mosaics—negatively affects shredders, resulting in reduced abundance and taxonomic richness (Faria et al., 2021; Luiza-Andrade et al., 2023). We also hypothesize that shredder biomass may remain stable or even increase in more impacted environments due to shifts in community dominance (Tonin et al., 2014; Uhler et al., 2021).

## Materials and methods

### Study area

**Fig. 1:**
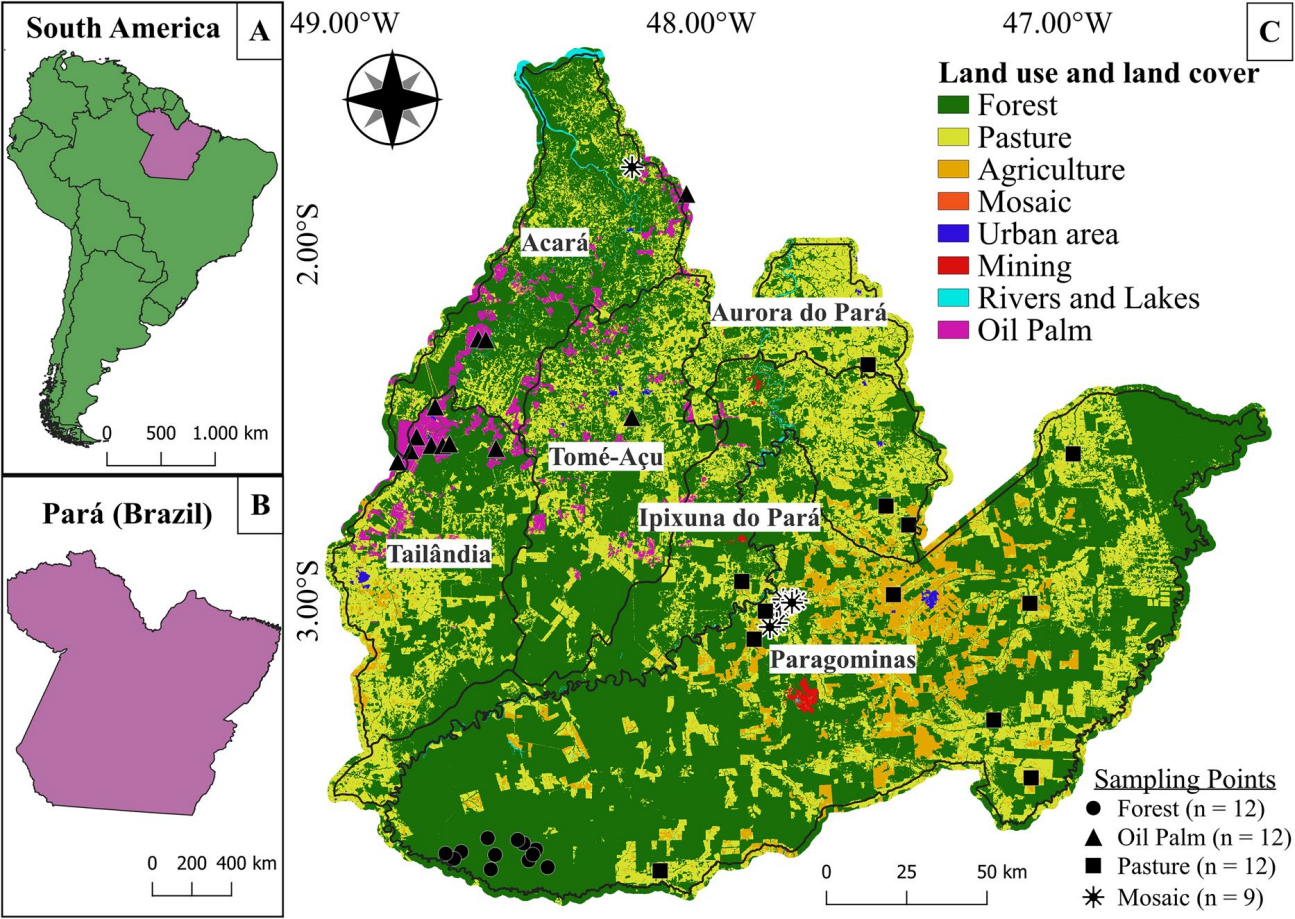
Distribution of the 45 sampled streams in the Acará and Capim river basins in the municipalities of Tailândia, Tomé-Açu, Ipixuna do Pará, Paragominas, Acará, and Aurora do Pará, Pará, Brazil

Sampling was conducted in 45 first- to third-order streams (Strahler, 1957) across the Acará and Capim river basins in northeastern Pará, Brazil (Fig. 1). The sites were located within the municipalities of Tailândia, Tomé-Açu, Ipixuna do Pará, Paragominas, Acará, and Aurora do Pará and were surveyed between 2011 and 2017 during the dry season, between June and November of each year. The region has a humid tropical climate, with a mean annual temperature of 26 °C and a relative humidity of 85% (Luiza-Andrade et al., 2017). The natural vegetation is a dense ombrophilous forest, which is now largely degraded. Over the past 15 years, the region has lost approximately 9% of its forest cover, while soybean cultivation has expanded most rapidly, increasing by approximately 1,073% (MapBiomas, 2025).

### Sampling design

**Fig. 2:**
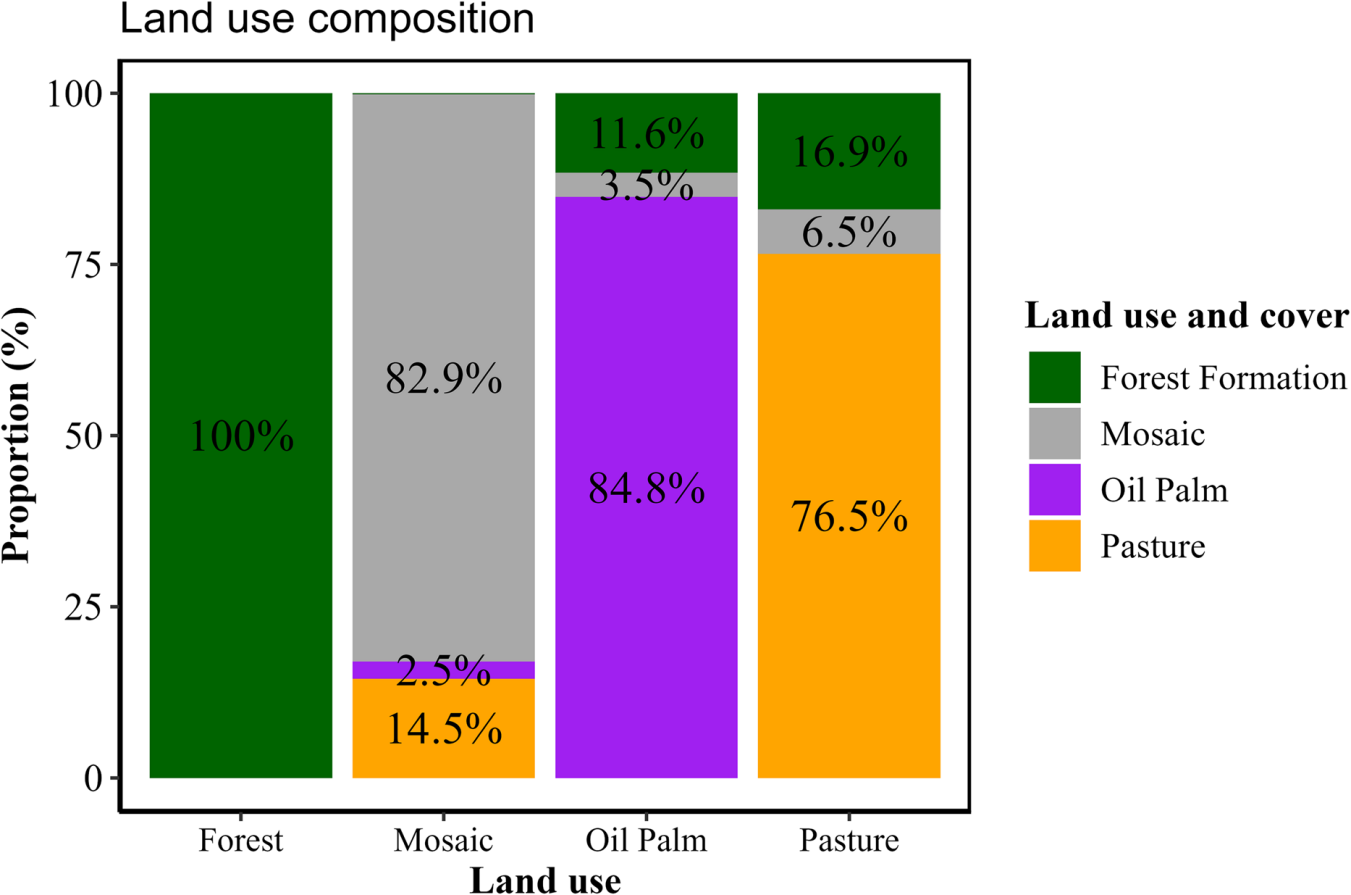
Mean relationships among stream categories by land use within the sampled catchment

Streams were categorized according to the dominant land-use and land-cover type (> 60%) in their catchments (Fig. 2). Forest cover data were obtained from the MapBiomas platform (collection 8.1). In each region, streams classified as “Forests” were dominated by forest formations. The “Pasture” sites were dominated by livestock pasture, whereas the “Palm” sites were dominated by oil palm monoculture. The “Mosaic” sites included agricultural landscapes with mixed crop and livestock uses and less than 25% forest cover. The delineation of the sampled stream catchments was conducted via a Digital Elevation Model (DEM) from the TOPODATA project of the *Instituto Nacional de Pesquisas Espaciais* (INPE). Coordinates were obtained at section “A” of each stream, following the sampling protocol of Callisto et al. (2014). Catchment delineation was then processed via the “*r.watershed*” algorithm in *QGIS* (version 3.34.9).

### Biological and limnological variables

A 150 m transect was established in each stream for the collection of biological samples and the measurement of environmental variables. This transect was divided into 11 cross-sections, labeled from downstream to upstream (A-K), creating 10 longitudinal sections spaced 15 m apart (Callisto et al., 2014; Luiza-Andrade et al., 2017). The environmental variables were measured at the beginning of each cross-section (Callisto et al., 2014). The variables assessed included the percentage of organic matter in the streambed (leaf litter, wood, macrophytes, roots, and algae), canopy cover, substrate type, riparian vegetation cover (visual estimation), human influence, and physicochemical and hydrological parameters (pH, temperature, dissolved oxygen, and flow).

Each longitudinal section was further subdivided into three 5 m segments, of which the first two were used for EPT collection. Samples were taken with an entomological drag net that was 18 cm in diameter and with 250 µm mesh (Faria et al., 2017; Luiza-Andrade et al., 2017). The biological material was sorted in the field via plastic trays and entomological tweezers and subsequently preserved in 85% ethanol (Faria et al., 2017; Luiza-Andrade et al., 2017). Quantification and identification at the genus level were conducted in the laboratory with the taxonomic keys of Hamada et al. (2014) and Domínguez et al. (2006).

### Classification of functional feeding groups (FFGs)

Ephemeroptera, Plecoptera, and Trichoptera were classified into functional feeding groups (FFGs) according to the scheme proposed by Merritt and Cummins (2019). To calculate abundance, richness, and biomass, as well as the relative proportion of shredders across land-use types, we followed the shredder definition of Houghton (2021) and Santos et al. (2024). For shredders, we considered the main representatives of each order, selecting the taxa with the highest affinity for shredding.

### Data analysis

The dry biomass (mg) of shredders was estimated via allometric equations from the literature that relate body mass to body length for each EPT genus (Dekanová et al., 2022; Paciência, 2012) (Table 1). Biomass estimation based on individual body size is widely used because it is fast, practical, and preserves specimens for future analyses, unlike traditional gravimetric approaches (Mährlein et al., 2016). In addition, due to the influence of ethanol preservation on individual weight and the variation in storage time among samples, the indirect biomass quantification method was the most reliable option for estimating the average productivity of streams under different land-use types (Leuven et al., 1985; Mährlein et al., 2016). Therefore, in this study, we selected the basic length-mass allometric model using constant values specifically developed for EPT insects from the Neotropical region, including the genera represented in our dataset, which provides more accurate biomass estimates than generalized or global models (Benke et al., 1999; Paciência, 2012).

**Table 1:** Allometric equations used to calculate the biomass of each genus

**Order**	**Family**	**Genus**	***a***	***b***	**Equation**	**Reference**
**Ephemeroptera**	Leptophlebiidae	*Fittkaulus*	−5.294	2.618	ln(DM) = ln(*a*) + *b* * ln(x^*^)	(Dekanová et al., 2022; Paciência, 2012)
**Plecoptera**	Perlidae	*Anacroneuria*	−1.789	2.555		
**Trichoptera**	Calamoceratidae	*Phylloicus*	−1.844	2.075		
	Leptoceridae	*Nectopsyche*	−1.73	2.126		
	Leptoceridae	*Triplectides*	−1.712	2.109		

^*^x = body length (cm)

The dry mass (DM) of each taxon was calculated via genus-specific constants (*a* and *b*) and the mean length of each genus (*x*). The total biomass of each genus (bg) was obtained by multiplying DM by the abundance of the respective genus $(bgx = DM \times Nx)$, considering the treatments separately. The total biomass per stream (bT) was then calculated as the sum of the biomass of each genus $(bT = \sum bgn$).

To evaluate how shredders respond to different land-use types, a one-factor ANOVA was performed, with richness, abundance, and biomass considered separately as response variables. Biological data were log-transformed (log(*x* + 1)) to reduce asymmetry among values (Legendre & Legendre, 2012). The assumptions of homogeneity of variances (Levene’s test, *p* > 0.05) and data normality (P-Plot) were verified beforehand. When significant differences were detected (*p* < 0.05), Tukey’s post hoc test was applied. The proportion of shredders was calculated from their abundance in each land-use type.

To identify the environmental variables influencing the distribution of shredder genera across different land-use categories, a Redundancy Analysis (RDA) was conducted with the selected environmental variables, using the *forward.sel* function (*p*-value < 0.05) (Dray et al., 2023). The relatively high abundances in each stream were Hellinger transformed to reduce the effect of dominance within the community, and the environmental variables were standardized via the *z* score method (Legendre & Legendre, 2012; Lima et al., 2022). All analyses were conducted in *R* software (version 4.4.0) through the *RStudio* interface (*v.*2025.05.0) via the “*stats*” (R Core Team, 2024), “*vegan*” (Oksanen et al., 2024), and “*adespatial*” packages (Dray et al., 2023).

## Results

A total of 775 shredder insects belonging to five genera of the orders EPT were analyzed (Table 2). We detected a significant effect of land use on shredder abundance (*F*_(3, 41)_ = 5.467; *p* = 0.003).

**Table 2:** Shredder abundance by dominant land use type

Abundance				
Genus	Oil palm	Forest	Mosaic	Pasture
*Anacroneuria*	87	58	9	12
*Fittkaulus*	0	0	0	24
*Nectopsyche*	0	8	0	1
*Phylloicus*	130	142	87	18
*Triplectides*	80	79	20	20
**Total**	**297**	**287**	**116**	**75**

**Fig. 3:**
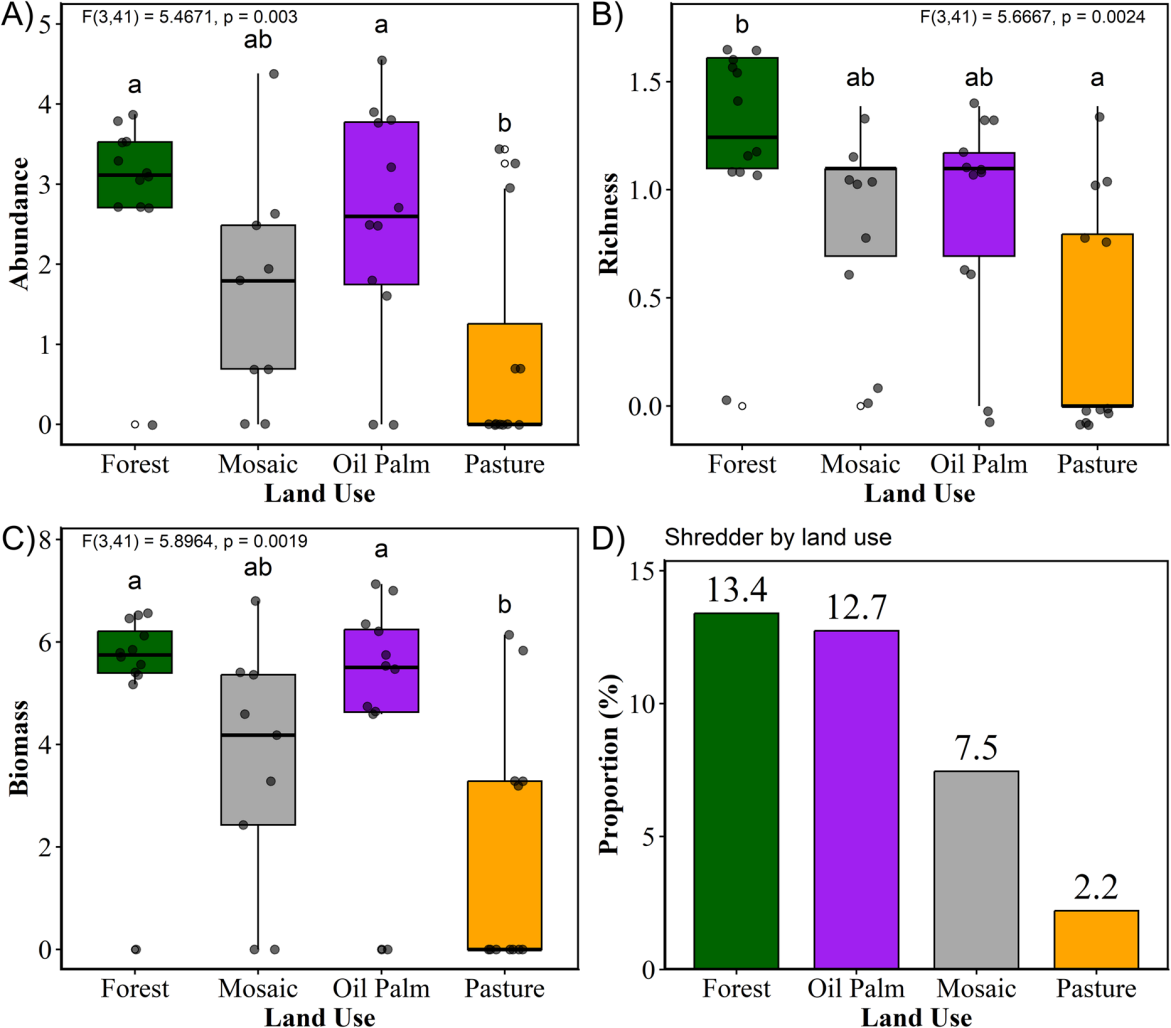
**A–D** Ecological responses of shredders in eastern Amazon streams across different land-use types. **A** Mean shredder abundance and standard deviation (SD) of shredders; **B** mean shredder richness and standard deviation (SD) of shredders; **C** mean dry biomass (mass − length) and standard deviation (SD) of shredders; **D** proportion of shredders per land-use type. The land-use categories are indicated by color: forest (green), mosaic (gray), oil palm (purple), and pasture (orange). Different letters represent statistically significant differences between treatments according to Tukey’s test

We detected a significant effect of land use on shredder abundance (*F*_(3, 41)_ = 5.467; *p* = 0.003). Compared with the pasture streams, the oil palm and forest streams presented significantly greater abundances (Fig. 3a). On average, the forest streams contained 17.6 more individuals than did the pasture streams (Tukey = 0.003), whereas the oil palm streams contained 18.5 more individuals (Tukey = 0.026). No significant differences, however, were detected between forest areas and oil palm (Tukey = 0.865) or mosaic streams (Tukey = 0.128). Similarly, mosaic streams did not differ significantly from oil palm (Tukey = 0.432) and pasture (Tukey = 0.631) streams.

For richness, land-use type had a significant effect, with different land-use and cover categories influencing the richness of shredder EPT (*F*_(3,41)_ = 5.666, *p* = 0.002). On average, the forest treatment had two more shredder species than did the pasture treatment (Tukey = 0.001) (Fig. 3b). No significant differences were observed among the other areas (Tukey_forest/mosaic_ = 0.191; Tukey_forest/oil palm_ = 0.394). Oil palm, mosaic, and pasture streams did not differ significantly from each other (Tukey_Oil Palm/Mosaic_ = 0.942; Tukey_Oil Palm/Pasture_ = 0.077; Tukey_Mosaic/Pasture_ = 0.317).

Compared with forest streams, there was significant variation in biomass among the treatments (*F*_(3,41)_ = 5.896, *p* = 0.002) (Table 3). Forest streams did not differ from oil palm (Tukey = 0.916) or mosaic streams (Tukey = 0.272). However, pasture streams had lower biomass than did forest areas, with an average difference of 286.7 mg (Tukey = 0.002), and compared with oil palm streams, with an average difference of 303.85 mg (Tukey = 0.012) (Fig. 3c). Mosaic streams showed no significant differences from oil palm (Tukey = 0.607) or pasture (Tukey = 0.301). The relative composition of shredders across land-use types followed similar patterns: the highest proportion was observed in forest streams, intermediate values in oil palm and mosaic, and the lowest in pasture (Fig. 3d).

**Table 3:** Shredder mean length and biomass per individual and per land-use type

Genus	Mean length (mm)	Individual biomass (mg)	Oil palm	Forest	Mosaic	Pasture
*Anacroneuria*	6.3	12.90	1122.41	748.27	116.11	154.81
*Fittkaulus*	5.5	0.61	0.00	0.00	0.00	14.61
*Nectopsyche*	7.5	8.72	0.00	69.79	0.00	8.72
*Phylloicus*	6.3	10.35	1345.42	1469.61	900.39	186.29
*Triplectides*	10.5	25.71	2057.15	2031.44	514.29	514.29
		**Total**	**4524.97**	**4319.11**	**1530.79**	**878.73**

**Fig. 4:**
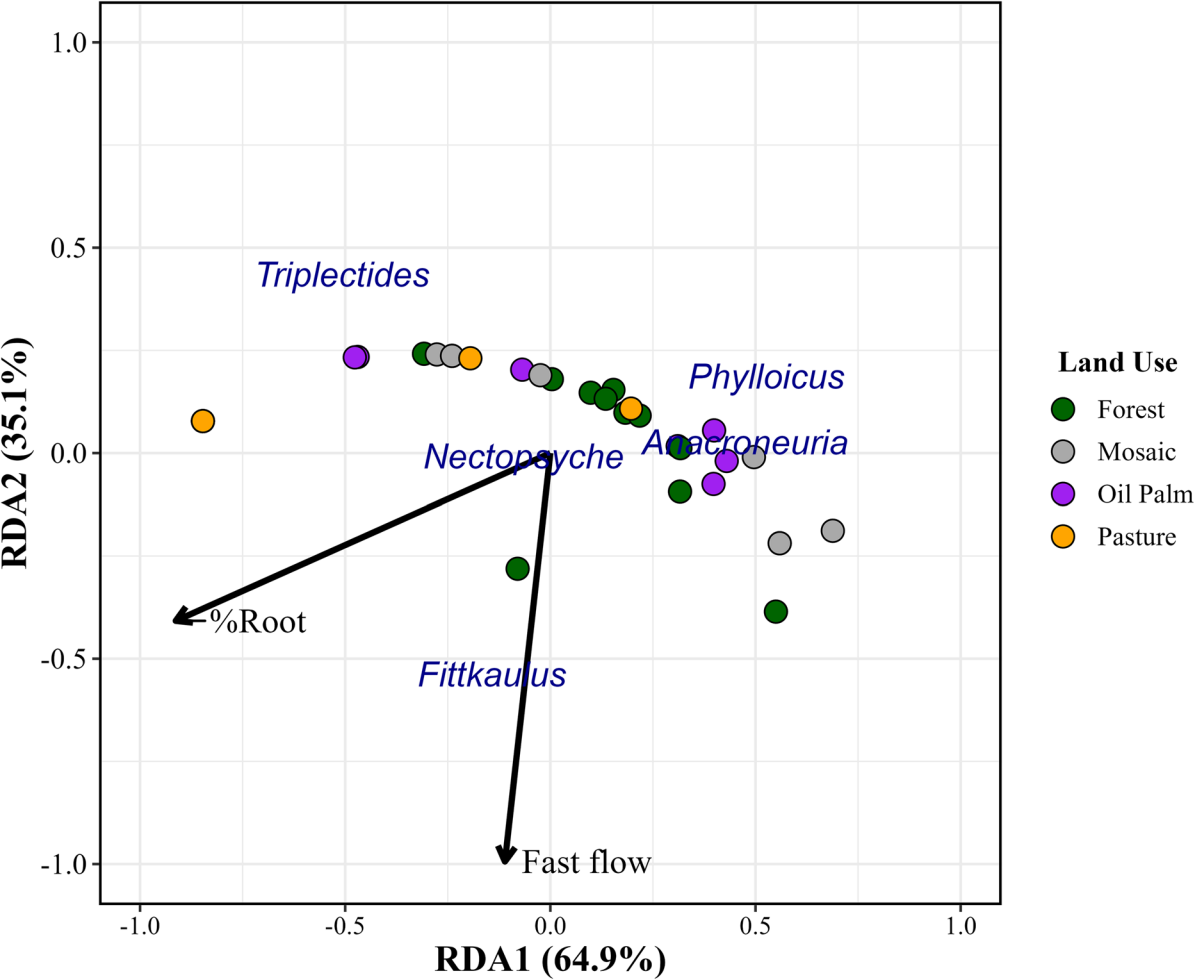
Relationships between environmental predictors and shredders through RDA in streams of the Capim and Acará River Basins, Pará, Brazil. The circles are colored according to the dominant land-use type

The first two axes of the RDA explained 34% of the variation in shredder composition (*F* = 9.22, adjusted *R*^2^ = 0.34, *p* = 0.001) (Fig. 4). Among the selected variables, only fast flow and root (%) were responsive to the distribution of shredder genera (*p* = 0.001).

The first axis (64.9%) represented a gradient of exposed root concentration, with higher percentages of roots negatively associated with the axis and predominated by points from pasture and mosaic streams (Table 4). In contrast, forest and oil palm streams were positioned positively along the axis, indicating lower presence of exposed roots. The second axis (35.1%) was structured primarily by fast flow, which was negatively associated with the axis and especially influenced streams located in forest, oil palm, and pasture catchments (Table 4).

**Table 4:** Contribution of environmental predictors to shredder abundance in streams of the Capim and Acará River Basins, Pará, Brazil (RDA axes 1 and 2)

Environmental predictor	RDA1	RDA2
Fast flow	−0.110	**−0.993**
%Root	**−0.913**	−0.407

Bold values indicate the strongest contribution of each predictor to each RDA axis

Among shredders, *Phylloicus*, *Anacroneuria*, and *Triplectides* were negatively associated with fast flow, whereas *Nectopsyche* and *Fittkaulus* showed a positive association. In addition, *Anacroneuria* and *Phylloicus* were more frequent in streams with a lower percentage of exposed roots, while *Triplectides* occurred in streams with higher concentrations of exposed roots.

## Discussion

Our hypothesis that shredder abundance, richness, and biomass are influenced by multiple land-use types was supported, as forest streams presented higher shredder metrics than did pasture streams. In headwaters (first- to third-order) or forested streams, riparian vegetation limits the primary productivity of algae and macrophytes, as the light reaching these systems is insufficient (Wallace et al., 1997; Rugenski et al., 2017, Chapter 28, p. 83; Graça et al., 2018; Albrecht et al., 2021). Consequently, allochthonous organic matter becomes the main source of energy and carbon in these ecosystems (Firmino et al., 2021; Vannote et al., 1980; Wallace et al., 1997). The reduction of allochthonous inputs due to deforestation alters the chemical and physical characteristics of streams, negatively affecting aquatic insect communities in the eastern Amazon and resulting in the loss of more sensitive genera and a decrease in taxonomic richness (Faria et al., 2021; Luiza-Andrade et al., 2023). In terms of dry biomass and shredder abundance, oil palm and forest streams differed significantly from pasture streams. In pastures, the analyses confirmed a loss of diversity, abundance, and individual biomass as a consequence of converting forested land to anthropogenic uses (Sonoda, 2025).

The EPT group is widely used in biomonitoring studies because of its broad distribution in aquatic environments and its plasticity in occupying different habitats (Akamagwuna et al., 2019, 2021; Bacca et al., 2023; Brasil et al., 2022). In addition, EPT taxa can be classified into FFGs based on morphological and behavioral traits related to feeding (Ramirez & Gutiérrez-Fonseca, 2014). The different types of organic matter consumed in streams—fine particulate organic matter (FPOM), coarse particulate organic matter (CPOM), prey, algae, and biofilms—are associated with distinct morphological adaptations, particularly in mouthpart structures (Cummins, 1973; Usseglio-Polatera et al., 2000; Poff et al., 2006; Gonçalves et al., 2014, Chapter 6, p. 89). The principal FFGs include collectors (both gatherers and filterers), shredders, predators, and scrapers (Cummins, 1973; Usseglio-Polatera et al., 2000; Poff et al., 2006; Gonçalves et al., 2014, Chapter 6, p. 89; Houghton, 2021).

Shredders are particularly important, as they convert CPOM into FPOM, accelerating leaf litter decomposition and making resources available to other organisms (Gonçalves et al., 2014, Chapter 6, p. 89; Sena et al., 2020). Their presence is influenced by leaf litter palatability, which depends on the decomposition stage, microbial conditioning, availability, and leaf quality (Kikuchi & Uieda, 2005; Martínez et al., 2013; Moretti et al., 2020; Silveira et al., 2006). Thus, changes in the quality of leaf litter entering streams can directly affect decomposition rates and ecosystem functioning (Firmino et al., 2021). Moreover, shredders are key agents of leaf litter decomposition because of their large biomass and body size, and their presence is therefore proportional to the decomposition rate of stream leaf litter (Gonçalves et al., 2014, Chapter 6, p. 89; Sena et al., 2020). Their close relationship with environmental gradients indicates that shredder loss is associated mainly with reduced inputs from riparian vegetation due to vegetation simplification (Brand & Miserendino, 2015; Serpa et al., 2020).

Compared with forested streams, agricultural streams presented the lowest shredder richness, suggesting that reduced resource availability affects organisms dependent on allochthonous energy inputs. Similarly, previous studies, such as Lima et al. (2022), have shown that shredders and collectors tend to be more strongly associated with forested streams, where allochthonous resource input is higher. Furthermore, it is well established that stream integrity and water quality are directly linked to surrounding vegetation (Tanaka et al., 2015). Consequently, vegetation homogenization and loss compromise stream integrity, leading to decreased aquatic community diversity and changes in microclimate, substrate, and nutrient inputs (Arce et al., 2023; Paiva et al., 2017).

In addition to the loss of organic matter (OM), riparian vegetation can influence organism distribution. Shredders preferentially consume more palatable, high-quality resources, which are more nutritious and attractive due to their lower C:N ratios and structural components, which vary according to plant type (Fernandes et al., 2015; Firmino et al., 2021; Martínez et al., 2013). This pattern may reflect shredder preference for native vegetation, which typically presents higher quality and palatability (Boyero et al., 2012; Pelizari et al., 2022) or results from the chemical composition of plants affecting microbial conditioning and influencing their feeding preference (Firmino et al., 2021; Kikuchi & Uieda, 2005). In line with this, forest cover directly influences soil chemistry, providing more nutrients and microorganisms to leaves (Santorufo et al., 2021). Consequently, when microbial activity is reduced, leaf conditioning is impaired, leading to slower decomposition and lower carbon and nitrogen concentrations, which classify the material as low quality (Firmino et al., 2021; Santorufo et al., 2021).

Furthermore, plant types may contain refractory, secondary, or toxic compounds that protect leaves against parasitism and herbivory, such as phenolic compounds (Graça et al., 2001; Oliveira & Nessimian, 2010; Gonçalves et al., 2014, Chapter 6, p. 89). These chemical traits inhibit microbial activity on leaves and consequently affect shredder palatability (Graça et al., 2001; Oliveira & Nessimian, 2010; Kochi et al., 2010; Gonçalves et al., 2014, Chapter 6, p. 89; Moretti et al., 2020). Therefore, shredders that select leaves according to palatability and quality may be affected in impacted streams because of the reduced availability of allochthonous resources caused by deforestation (Boyero et al., 2012; Firmino et al., 2021; Pelizari et al., 2022).

Biomass, in addition to being important for assessing ecosystem structure and energy flow, also reflects environmental productivity (Cummins et al., 2022; Lynch, 2024). Thus, although the richness of pasture streams may not differ from that of oil palm streams, they differ in terms of biomass and abundance. Moreover, a comparison of these attributes revealed that biomass variation increases in pasture streams, whereas it decreases in oil palm streams, despite abundance showing the opposite pattern. This highlights how land-use type influences environmental productivity, reflected in the allometric measures of individuals, regardless of abundance. This supports the statement by Uhler et al. (2021), who argued that an increase in large-bodied species may influence biomass but does not necessarily affect the total number of species. In pasture streams, although Tonin et al. (2014) suggest that large-bodied shredders could help sustain overall biomass even when abundance decreases, our results did not reflect this expected pattern or our initial hypothesis. Instead, the marked reduction in forest cover was accompanied by declines in both shredder abundance and biomass. The observed decline in biomass in degraded areas highlights the negative influence of land-use change on stream productivity. This result supports previous studies that associate higher biomass with intact environments, given the strong dependence of shredders on allochthonous resources (Houghton, 2021).

The impact of deforestation and alterations in riparian vegetation is reflected in the variation in biomass in pasture streams. Among the treatments, only the pasture streams presented a significant reduction in biomass, with a lower mean biomass than the forest and oil palm streams did. This finding indicates that the greatest impact on local productivity occurred in streams with relatively high deforestation rates and no forest replacement. This pattern may also explain the absence of significant differences between pasture streams and those with less than 60% forest cover, classified as mosaic. Despite the lack of differences among environments with lower vegetation cover, mosaic streams sustained sufficient biomass to show no significant differences compared with forest and oil palm streams. This pattern can be explained by the *Intermediate Disturbance Hypothesis*, proposed by Connell (1978), which states that in environments subjected to moderate levels of disturbance, species that previously had low dispersal and growth rates are able to establish more successfully, resulting in higher diversity compared with more highly disturbed environments. Forested streams had a greater proportion of shredders, which are typically associated with greater availability of organic matter (Houghton, 2021). As primary consumers in forested streams, their higher proportion is explained by their dependence on organic matter inputs (Brasil et al., 2014; Oliveira & Nessimian, 2010).

RDA revealed that the composition of the analyzed genera was strongly structured along an environmental gradient represented by the percentage of exposed roots (%Root) and stream flow. The first axis represented higher percentages of root cover associated with pasture and mosaic streams, a pattern also observed by Luiza-Andrade et al*.* (2022) in impacted environments. This pattern is consistent with the advance of erosive processes in human-impacted streams, where the removal of riparian vegetation favors bank instability (Luiza-Andrade et al., 2022; Santorufo et al., 2021). The second axis was structured by fast flow, was negatively associated with the axis, and influenced mainly streams located in forest, oil palm, and pasture catchments.

Although the RDA did not directly include variables related to OM quality or microbial activity, it is plausible that vegetation cover and flow dynamics indirectly influence detritus conditioning, affecting its quality and availability (Canhoto et al., 2013; Richardson & Neill, 1991; Santorufo et al., 2021). As stated by Rolls et al. (2012), slow flows associated with more stable banks favor the deposition and accumulation of particulate organic matter, creating microhabitats suitable for shredder activity. These results suggest that alterations in riparian cover and hydrological regimes influence not only the physical structure of streams but also the ecological processes underlying decomposition and energy flow in aquatic systems (Rolls et al., 2012).

The response of shredders to these variables suggests distinct ecological strategies among taxa. Among shredders, *Phylloicus*, *Anacroneuria*, and *Triplectides* were negatively associated with fast flow, indicating a preference for low-current environments where detritus retention and substrate stability favor organic matter fragmentation (Rolls et al., 2012). Whereas *Nectopsyche* and *Fittkaulus* were positively associated, suggesting greater tolerance to unstable hydrodynamic conditions and, as shredders, a particular preference for more aerated streams with enhanced leaf conditioning, as observed by Canhoto et al. (2013). In addition, *Anacroneuria* and *Phylloicus* were more common in streams with a lower percentage of exposed roots, whereas *Triplectides* occurred in streams with higher concentrations of exposed roots, possibly exploiting these structures as refuges or zones of resource accumulation (Luiza-Andrade et al., 2022). The relationships between the explanatory variables and the observed patterns align with evidence that riparian vegetation integrity directly affects the hydrological and physical conditions of lotic ecosystems (Santorufo et al., 2021). In preserved forest streams, practices such as active restoration with native species contribute to increased infiltration, water retention, and soil stability (Brizzi et al., 2018; Rodrigues & Torres, 2025). Conversely, the removal of vegetation cover in pasture or urban land-use streams intensifies erosion and reduces the functionality of riparian zones (Gomes et al., 2018).

Despite this, 66% of the variation in genera distribution remains unexplained, indicating that other factors influence shredder distribution, even indirectly. Given that the distribution of individuals was more strongly associated with streambed structure, it is plausible to suggest that such structural modification indirectly affects shredders by favoring predators—since the substrate is also used as protection from attacks (Hamada et al., 2014)—or by hindering foraging, creating physical obstacles and limiting the availability of organic matter on the streambed. To further investigate these patterns, it would be useful to perform an RDA including more specific environmental variables related to streambed physical structure, or to conduct correlation analyses exploring biological relationships between shredders and their main predators.

The loss of shredder biomass and diversity highlights how anthropogenic alterations, such as deforestation, destabilize aquatic communities from the base of the food web (Yule et al., 2010). As primary consumers, shredders are linked to predators (both vertebrates and invertebrates), whose presence depends on prey availability, thereby affecting higher trophic levels through a cascading effect (Brasil et al., 2014; Oliveira & Nessimian, 2010). Additionally, since shredders contribute to carbon and nutrient cycling through leaf litter decomposition in streams, their long-term loss may alter community composition or slow biogeochemical processes, such as carbon cycling (Sonoda, 2025).

## Conclusion

We demonstrated that the replacement of forested streams with anthropogenic land significantly reduces the abundance, richness, and biomass of shredder EPT taxa.

Our study integrated multiple biological metrics, directly linking shredder community structure with land use and land cover. Including biomass as a complementary metric revealed differences in ecological productivity that would not be evident from individual counts or species richness alone, contributing to a more robust assessment of environmental impact and energy flow. The reduction in shredder diversity, abundance, and biomass compromises not only the base of the food web but also nutrient dynamics and the ecological processes associated with decomposition and carbon cycling.

Despite the ecological importance of aquatic insects, studies addressing their environmental preferences and responses to gradients of anthropogenic alterations in the Amazon remain scarce. Our results highlight the urgency of expanding research efforts and strengthening public policies aimed at conserving and monitoring aquatic ecosystems. Biological indices based on macroinvertebrates, such as shredders, appear promising for detecting environmental degradation and guiding management actions.

The preservation and restoration of riparian vegetation are fundamental to maintaining ecological functions in lotic ecosystems and achieving global sustainability targets, particularly within the framework of the UN Sustainable Development Goals. This study provides evidence reinforcing the need to protect aquatic biodiversity and associated ecosystem services, especially in regions of high environmental and ecological importance, such as the Amazon.

## Data Availability

The datasets used and analysed during the current study are available from the corresponding author upon reasonable request.

## References

[CR1] Akamagwuna, F. C., Mensah, P. K., Nnadozie, C. F., & Odume, O. N. (2019). Trait-based responses of Ephemeroptera, Plecoptera, and Trichoptera to sediment stress in the Tsitsa River and its tributaries, Eastern Cape, South Africa. *River Research and Applications,**35*(7), 999–1012. 10.1002/rra.3458 10.1007/s10661-019-7846-9 31650234

[CR2] Akamagwuna, F. C., Ntloko, P., Edegbene, A. O., et al. (2021). Are Ephemeroptera, Plecoptera and Trichoptera traits reliable indicators of semi-urban pollution in the Tsitsa River, Eastern Cape Province of South Africa? *Environmental Monitoring and Assessment,**193*, Article 309. 10.1007/s10661-021-09093-z 33913034 10.1007/s10661-021-09093-z

[CR3] Albrecht, M., Reis, A., Neres-Lima, V., & Zandonà, E. (2021). Isótopos estáveis e outras ferramentas em estudos tróficos de peixes em riachos tropicais. *Oecologia Australis,**25*(2), 283–300. 10.4257/oeco.2021.2502.05

[CR4] Allen, D. C., Larson, J., Murphy, C. A., Garcia, E. A., Anderson, K. E., Busch, M. H., et al. (2024). Global patterns of allochthony in stream–riparian meta-ecosystems. *Ecology Letters*. 10.1111/ele.14401 10.1111/ele.14401 38468439

[CR5] Arce, A. P., Kail, J., Tasser, E., et al. (2023). The effect of riparian forest on landscape connectivity for the EPT community across European regions. *Hydrobiologia*. 10.1007/s10750-023-05353-w

[CR6] Bacca, C., Cararo, R., Magro, D., & de Rezende, S. (2023). Macroinvertebrados bentônicos como indicadores da qualidade da água em riachos de campos e florestas de altitudes. *Revista Espinhaço, 11*(1). 10.5281/zenodo.7647310

[CR7] Benke, A. C., Huryn, A. D., Smock, L. A., & Wallace, J. B. (1999). Length–mass relationships for freshwater macroinvertebrates in North America with particular reference to the southeastern United States. *Journal of the North American Benthological Society,**18*(3), 308–343. 10.2307/1468447

[CR8] Boyero, L., Barmuta, L. A., Ratnarajah, L., Schmidt, K., & Pearson, R. G. (2012). Effects of exotic riparian vegetation on leaf breakdown by shredders: A tropical–temperate comparison. *Freshwater Science,**31*(2), 296–303. 10.1899/11-103.1

[CR9] Brand, C., & Miserendino, M. L. (2015). Testing the performance of macroinvertebrate metrics as indicators of changes in biodiversity after pasture conversion in Patagonian mountain streams. *Water, Air, & Soil Pollution,**226*, Article 370. 10.1007/s11270-015-2633-x

[CR10] Brasil, L. S., et al. (2022). Insetos aquáticos bioindicadores de mudanças de uso da terra no Pará, Brasil: Evidências e perspectivas. *Oecologia Australis,**26*(3), 424–444. 10.4257/oeco.2022.2603.03

[CR11] Brasil, L. S., Juen, L., Batista, J. D., Pavan, M. G., & Cabette, H. S. (2014). Longitudinal distribution of the functional feeding groups of aquatic insects in streams of the Brazilian Cerrado savanna. *Neotropical Entomology,**43*(5), 421–428. 10.1007/s13744-014-0234-9 27193952 10.1007/s13744-014-0234-9

[CR12] Brizzi, R. R., de Souza, A. P., & da Costa, A. J. S. T. (2018). Relação entre a infiltração da água nos solos e a estabilidade dos agregados em sistemas de manejos diferentes na bacia hidrográfica do rio São Romão - Nova Friburgo / RJ. *Caminhos De Geografia,**19*(67), 304–321. 10.14393/Hygeia196720

[CR13] Callisto, M., et al. (2014). *Condições ecológicas em bacias hidrográficas de empreendimentos hidrelétricos* (1st ed). CEMIG. Retrieved March 15, 2024, from https://www.cemig.com.br/wp-content/uploads/2020/07/Indice_de_Integridade_Biotica.pdf

[CR14] Canhoto, C., Calapez, R., Gonçalves, A. L., & Moreira-Santos, M. (2013). Effects of Eucalyptus leachates and oxygen on leaf-litter processing by fungi and stream invertebrates. *Freshwater Science,**32*(2), 411–424. 10.1899/12-062.1

[CR15] Connell, J. H. (1978). Diversity in tropical rain forests and coral reefs. *Science,**199*(4335), 1302–1310. 10.1126/science.199.4335.1302 17840770 10.1126/science.199.4335.1302

[CR16] Cummins, K. W. (1973). Trophic relations of aquatic insects. *Annual Review of Entomology,**18*, 183–206. 10.1146/annurev.en.18.010173.001151

[CR17] Cummins, K. W., Wilzbach, M., Kolouch, B., & Merritt, R. (2022). Estimating macroinvertebrate biomass for stream ecosystem assessments. *International Journal of Environmental Research and Public Health,**19*(6), Article 3240. 10.3390/ijerph19063240 35328929 10.3390/ijerph19063240 PMC8955383

[CR18] Dekanová, V., Venarsky, M. P., & Bunn, S. E. (2022). Length–mass relationships of Australian aquatic invertebrates. *Austral Ecology,**47*, 120–126. 10.1111/aec.13077

[CR19] DeWalt, R. E., & Ower, G. D. (2019). Ecosystem services, global diversity, and rate of stonefly species descriptions (Insecta: Plecoptera). *InSects,**10*(4), Article 99. 10.3390/insects10040099 30959938 10.3390/insects10040099 PMC6523762

[CR20] Domínguez, E., Molineri, C., Pescador, M. L., Hubbard, M. D., & Nieto, C. (2006). Ephemeroptera of South America. In J. Adis, J. R. Arias, G. Rueda-Delgado, & K. M. Wantzen (Eds.),* Aquatic biodiversity in Latin America**(ABLA) *(Vol. 2, pp. 1–646). Pensoft.

[CR21] Dray, S., Bauman, D., Blanchet, G., Borcard, D., Clappe, S., Guénard, G., Jombart, T., Larocque, G., Legendre, P., Madi, N., & Wagner, H. H. (2023). *Adespatial: multivariate multiscale spatial analysis* (R package version 0.3–23). https://CRAN.R-project.org/package=adespatial

[CR22] Faria, A. P. J., Ligeiro, R., Callisto, M., & Juen, L. (2017). Response of aquatic insect assemblages to the activities of traditional populations in eastern Amazonia. *Hydrobiologia, 802*(1), 39–51. 10.1007/s10750-017-3238-8

[CR23] Faria, A. P. J., Paiva, C. K. S., Calvão, L. B., et al. (2021). Response of aquatic insects to an environmental gradient in Amazonian streams. *Environmental Monitoring and Assessment,**193*, Article 763. 10.1007/s10661-021-09553-6 34729664 10.1007/s10661-021-09553-6

[CR24] Fernandes, I., Duarte, S., Cássio, F., & Pascoal, C. (2015). Plant litter diversity affects invertebrate shredder activity and the quality of fine particulate organic matter in streams. *Marine and Freshwater Research,**66*, 449–458. 10.1071/MF14089

[CR25] Firmino, V. C., Brasil, L. S., Martins, R. T., et al. (2021). Litter decomposition of exotic and native plant species of agricultural importance in Amazonian streams. *Limnology,**22*, 289–297. 10.1007/s10201-021-00655-1

[CR26] Gomes, E., Jesus, E., Oliveira, N., Júnior, L., Cabral, F., & Resende, M. (2018). A nova legislação ambiental brasileira e seus efeitos sobre a reestruturação de nascentes e remanescentes florestais. *Pesquisa Florestal Brasileira*, 38. 10.4336/2018.pfb.38e201601309

[CR27] Gonçalves, J. F., Martins, R. T., Ottoni, B. M. P., & Couceiro, S. E. M. (2014). Uma visão sobre a decomposição foliar em sistemas aquáticos brasileiros. In N. Hamada, J. L. Nessimian, & R. B. Querino (Eds.), *Insetos aquáticos na Amazônia brasileira: Taxonomia, biologia e ecologia* (pp. 89–116). Editora do INPA. https://repositorio.inpa.gov.br/handle/1/36169

[CR28] Graça, M. A. S., Callisto, M., Barbosa, J. E. L., Firmiano, K. R., França, J., & Júnior, J. F. G. (2018). Top-down and bottom-up control of epilithic periphyton in a tropical stream. *Freshwater Science*. 10.1086/700886

[CR29] Graça, M., Cressa, C., Gessner, M., Feio, M., Callies, K. A., & Barrios, C. (2001). Food quality, feeding preferences, survival and growth of shredders from temperate and tropical streams. *Freshwater Biology,**46*, 947–957. 10.1046/j.1365-2427.2001.00729.x

[CR30] Hamada, N., Nessimian, J. L., & Querino, R. B. (2014). *Insetos aquáticos na Amazônia brasileira: Taxonomia, biologia e ecologia* (p. 724). Editora do INPA. https://repositorio.inpa.gov.br/handle/1/36169

[CR31] Houghton, D. C. (2021). A tale of two habitats: Whole-watershed comparison of disturbed and undisturbed river systems in northern Michigan (USA), based on adult Ephemeroptera, Plecoptera, and Trichoptera assemblages and functional feeding group biomass. *Hydrobiologia,**848*, 3429–3446. 10.1007/s10750-021-04579-w

[CR32] Juen, L., Cunha, E. J., Carvalho, F. G., Ferreira, M. C., Begot, T. O., Luiza-Andrade, A., Shimano, Y., Leão, H., Pompeu, P. S., & Montag, L. F. A. (2016). Effects of oil palm plantations on the habitat structure and biota of streams in Eastern Amazon. *River Research and Applications,**32*(10), 2081–2094. 10.1002/rra.3050

[CR33] Kikuchi, R. M., & Uieda, V. S. (2005). Composição e distribuição dos macroinvertebrados em diferentes substratos de fundo de um riacho no Município de Itatinga, São Paulo, Brasil. *Entomología y Vectores,**12*(2), 193–231. 10.1590/S0328-03812005000200006

[CR34] Kochi, K., Kagaya, T., & Kusumoto, D. (2010). Does mixing of senescent and green leaves result in nonadditive effects on leaf decomposition? *Journal of the North American Benthological Society,**29*(2), 454–464. 10.1899/08-190.1

[CR35] Legendre, P., & Legendre, L. (2012). *Numerical ecology *(3rd ed). (Developments in Environmental Modelling, Vol. 24). Elsevier.

[CR36] Leuven, R. S. E. W., Brock, T. C. M., & van Druten, H. A. M. (1985). Effects of preservation on dry- and ash-free dry weight biomass of some common aquatic macro-invertebrates. *Hydrobiologia,**127*, 151–159. 10.1007/BF00004193

[CR37] Lima, M., Firmino, V. C., Paiva, C. K. S., et al. (2022). Land use changes disrupt streams and affect the functional feeding groups of aquatic insects in the Amazon. *Journal of Insect Conservation,**26*, 137–148. 10.1007/s10841-022-00375-6

[CR38] Luiza-Andrade, A., Brasil, L. S., Benone, N. L., Shimano, Y., Farias, A. P. J., Montag, L. F., Dolédec, S., & Juen, L. (2017). Influence of oil palm monoculture on the taxonomic and functional composition of aquatic insect communities in eastern Brazilian Amazonia. *Ecological Indicators,**82*, 478–483. 10.1016/j.ecolind.2017.07.006

[CR39] Luiza-Andrade, A., Brasil, L. S., Torres, N. R., et al. (2020). Effects of local environmental and landscape variables on the taxonomic and trophic composition of aquatic insects in a rare forest formation of the Brazilian Amazon. *Neotropical Entomology,**49*, 821–831. 10.1007/s13744-020-00814-6 32946024 10.1007/s13744-020-00814-6

[CR40] Luiza-Andrade, A., Silva, R. R., & Juen, L. (2023). Contribution of rare genera of aquatic insects to functional diversity in streams with multiple land use in the Amazon. *Hydrobiologia,**850*, 21–38. 10.1007/s10750-022-05035-z

[CR41] Luiza-Andrade, A., Silva, R. R., Shimano, Y., et al. (2022). Niche breadth and habitat preference of Ephemeroptera, Plecoptera, and Trichoptera (Insecta) in streams in the Brazilian Amazon. *Hydrobiologia,**849*, 4287–4306. 10.1007/s10750-022-04987-6

[CR42] Lynch, M. (2024). The bioenergetic cost of building a metazoan. *Proceedings of the National Academy of Sciences of the United States of America*. 10.1073/pnas.2414742121 10.1073/pnas.2414742121 PMC11573499 39508768

[CR43] Mährlein, M., Pätzig, M., Brauns, M., et al. (2016). Length–mass relationships for lake macroinvertebrates corrected for back-transformation and preservation effects. *Hydrobiologia,**768*, 37–50. 10.1007/s10750-015-2526-4

[CR44] MapBiomas. (2025). *Projeto de mapeamento anual da cobertura e uso da terra no Brasil. *Retrieved March 15, 2024, from https://mapbiomas.org/.

[CR45] Martínez, A., Larrañaga, A., Pérez, J., Basaguren, A., & Pozo, J. (2013). Leaf-litter quality effects on stream ecosystem functioning: A comparison among five species. *Fundamental and Applied Limnology/archiv Für Hydrobiologie,**183*, 239–248. 10.1127/1863-9135/2013/0514

[CR46] Merritt, R. W., & Cummins, K. W. (2019). *An introduction to the aquatic insects of North America* (5th ed). Kendall/Hunt Publishing Company.

[CR47] Moretti, M. S., Becker, B., Kiffer Jr., W. P., da Penha, L. O., & Callisto, M. (2020). Eucalyptus leaves are preferred to cerrado native species but do not constitute a better food resource to stream shredders. *Journal of Arid Environments*, 181. 10.1016/j.jaridenv.2020.104221

[CR48] Morse, J. C., Frandsen, P. B., Graf, W., & Thomas, J. A. (2019). Diversity and ecosystem services of Trichoptera. *InSects,**10*(5), Article 125. 10.3390/insects10050125 31052441 10.3390/insects10050125 PMC6572163

[CR49] Oester, R., dos Reis Oliveira, P. C., Moretti, M. S., et al. (2023). Leaf-associated macroinvertebrate assemblage and leaf litter breakdown in headwater streams depend on local riparian vegetation. *Hydrobiologia,**850*(15), 3359–3374. 10.1007/s10750-022-05049-7 37397167 10.1007/s10750-022-05049-7 PMC10307707

[CR50] Oksanen, J., Simpson, G. L., Blanchet, F. G., Kindt, R., Legendre, P., Minchin, P. R., et al. (2024). *vegan: Community ecology package* (Version 2.6–8). 10.32614/CRAN.package.vegan

[CR51] Oliveira, A. L. H., & Nessimian, J. L. (2010). Spatial distribution and functional feeding groups of aquatic insect communities in Serra da Bocaina streams, southeastern Brazil. *Acta Limnologica Brasiliensia,**22*(4), 424–441. 10.4322/actalb.2011.007

[CR52] Paciência, G. P. (2012). *Avaliando os efeitos do tamanho do riacho, do tipo de mesohabitat e da estação do ano sobre a fauna de Ephemeroptera, Plecoptera e Trichoptera* [Doctoral dissertation, Universidade de São Paulo]. Biblioteca Digital de Teses e Dissertações da USP. 10.11606/T.59.2012.tde-05052013-121602

[CR53] Paiva, C. K. S., de Faria, A. P. J., Calvao, L. B., & Juen, L. (2017). Effect of oil palm on the Plecoptera and Trichoptera (Insecta) assemblages in streams of eastern Amazon.* Environmental monitoring and assessment, 189*(8), 393.. 10.1007/s10661-017-6116-y 10.1007/s10661-017-6116-y 28707254

[CR54] Peckarsky, B. L., & Lamberti, G. A. (2017). Invertebrate consumer–resource interactions. In F. R. Hauer, & G. A. Lamberti (Eds.), *Methods in Stream Ecology* (vol. 1, 3rd ed., pp. 379–398). Academic Press.

[CR55] Pelizari, G. P., et al. (2022). Leaf breakdown in a tropical stream: Comparison between the exotic Eucalyptus grandis and two native species. *Acta Limnologica Brasiliensia,**34*, Article e12. 10.1590/S2179-975X2321

[CR56] Poff, N., Olden, J., Vieira, N., Finn, D., Simmons, M., & Kondratieff, B. (2006). Functional trait niches of North American lotic insects: Traits-based ecological applications in light of phylogenetic relationships. *Journal of the North American Benthological Society,**25*, 730–755. 10.1899/0887-3593(2006)025(0730:FTNONA)2.0.CO;2

[CR57] Pozo, J., González, E., Díez, J. R., Molinero, J., & Elósegui, A. (1997). Inputs of particulate organic matter to streams with different riparian vegetation. *Journal of the North American Benthological Society,**16*(3), 602–611. 10.2307/1468147

[CR58] R Core Team. (2024). *R: A language and environment for statistical computing*. Vienna: R Foundation for Statistical Computing. Retrieved July 03, 2024, from https://www.R-project.org/

[CR59] Ramirez, A., & Gutiérrez-Fonseca, P. E. (2014). Functional feeding groups of aquatic insect families in Latin America: A critical analysis and review of existing literature. *Revista De Biología Tropical,**62*, 155–167. 10.15517/rbt.v62i0.15785 25189076 10.15517/rbt.v62i0.15785

[CR60] Rebora, M., Salerno, G., & Piersanti, S. (2019). Aquatic insect sensilla: Morphology and function. In Del-Claro, K, & Guillermo, R. Aquatic insects: *Behavior and ecology* (pp. 139–166). Springer Cham. 10.1007/978-3-030-16327-3_7

[CR61] Richardson, J. S., & Neill, W. E. (1991). Indirect effects of detritus manipulations in a montane stream. *Canadian Journal of Fisheries and Aquatic Sciences,**48*(5), 776–783. 10.1139/f91-093

[CR62] Rodrigues, V., & Torres, F. T. P. T. (2025). Influência em curto prazo de diferentes métodos de restauração florestal na disponibilidade e qualidade de nascentes na região sul de Minas Gerais. *UÁQUIRI - Revista Do Programa De Pós Graduação Em Geografia Da Universidade Federal Do Acre, **6*(1), 1–3. 10.29327/2463817.6.1-3

[CR63] Rolls, R. J., Leigh, C., & Sheldon, F. (2012). Mechanistic effects of low-flow hydrology on riverine ecosystems: Ecological principles and consequences of alteration. *Freshwater Science,**31*(4), 1163–1186. 10.1899/12-002.1

[CR64] Rugenski, A. T., Minshall, G. W., & Hauer, F. R. (2017). Riparian processes and interactions. In F. R. Hauer, & G. A. Lamberti (Eds.), *Methods in Stream Ecology* (vol. 2, 3rd ed., pp. 83–111). Academic Press.

[CR65] Santorufo, L., et al. (2021). Impact of anthropic activities on soil quality under different land uses. *International Journal of Environmental Research and Public Health,**18*(16), Article 8423. 10.3390/ijerph18168423 34444172 10.3390/ijerph18168423 PMC8393834

[CR66] Santos, N. B. B., Cruz, G. M., Monteles, J. S., et al. (2024). Database of immature stage traits of Ephemeroptera, Plecoptera, and Trichoptera (EPT) genera for the Amazon. *Aquatic Sciences,**86*, Article 35. 10.1007/s00027-024-01051-4

[CR67] Sena, G., Gonçalves Junior, J. F., Martins, R. T., Hamada, N., & Rezende, R. D. S. (2020). Leaf litter quality drives the feeding by invertebrate shredders in tropical streams. *Ecology and Evolution,**10*(16), 8563–8570. 10.1002/ece3.6169 32884640 10.1002/ece3.6169 PMC7452764

[CR68] Serpa, K. V., Kiffer, W. P., Borelli, M. F., et al. (2020). Niche breadth of invertebrate shredders in tropical forest streams: Which taxa have restricted habitat preferences? *Hydrobiologia,**847*, 1739–1752. 10.1007/s10750-019-04149-1

[CR69] Silveira, M. P., et al. (2006). Spatial and temporal distribution of benthic macroinvertebrates in a Southeastern Brazilian River. *Brazilian Journal of Biology,**66*(2b), 623–632. 10.1590/S1519-69842006000400006 10.1590/s1519-69842006000400006 16906294

[CR70] Sonoda, K. C. (2025). *Efeitos dos usos do solo sobre insetos de ambientes aquáticos brasileiros.* Embrapa. Retrieved August 11, 2025, from http://www.alice.cnptia.embrapa.br/alice/handle/doc/1173913

[CR71] Stanford, J. A., Alexander, L. C., & Whited, D. C. (2017). Riverscapes. In F. R. Hauer & G. A. Lamberti (Eds.), *Methods in stream ecology* (3rd ed., Vol. 1, pp. 3–20). Academic Press.

[CR72] Strahler, A. N. (1957). Quantitative analysis of watershed geomorphology. *Transactions of the American Geophysical Union,**38*, 913–920. 10.1029/TR038i006p00913

[CR73] Tanaka, M. O., et al. (2015). Redundância entre métricas da qualidade ambiental de riachos em paisagem agrícola. *Ambiente e Agua - an Interdisciplinary Journal of Applied Science,**10*(4), 832–846. 10.4136/ambi-agua.1665

[CR74] Tchakonté, S., Ajeagah, G. A., Camara, A. I., et al. (2015). Impact of urbanization on aquatic insect assemblages in the coastal zone of Cameroon: The use of biotraits and indicator taxa to assess environmental pollution. *Hydrobiologia,**755*, 123–144. 10.1007/s10750-015-2221-5

[CR75] Thorp, J. H., & Covich, A. P. (2010). *Ecology and classification of North American freshwater invertebrates* (3rd ed.). Academic Press.

[CR76] Tonin, A. M., Hepp, L. U., Restello, R. M., et al. (2014). Understanding of colonization and breakdown of leaves by invertebrates in a tropical stream is enhanced by using biomass as well as count data. *Hydrobiologia,**740*, 79–88. 10.1007/s10750-014-1939-9

[CR77] Uhler, J., Redlich, S., Zhang, J., et al. (2021). Relationship of insect biomass and richness with land use along a climate gradient. *Nature Communications,**12*, 5946. 10.1038/s41467-021-26181-3 10.1038/s41467-021-26181-3 PMC8511018 34642336

[CR78] United Nations. (2025). *Sustainable Development Goals.* United Nations. Retrieved January 20, 2025, from https://sdgs.un.org/goals

[CR79] Usseglio-Polatera, P., Bournaud, M., Richoux, P., & Tachet, H. (2000). Biological and ecological traits of benthic freshwater macroinvertebrates: Relationships and definition of groups with similar traits. *Freshwater Biology,**43*, 175–205. 10.1046/j.1365-2427.2000.00535.x

[CR80] Valente-Neto, F., Koroiva, R., Fonseca-Gessner, A. A., et al. (2015). The effect of riparian deforestation on macroinvertebrates associated with submerged woody debris. *Aquatic Ecology,**49*, 115–125. 10.1007/s10452-015-9510-y

[CR81] Vannote, R. L., et al. (1980). The River Continuum Concept. *Canadian Journal of Fisheries and Aquatic Sciences,**37*(1), 130–137. 10.1139/f80-017

[CR82] Wallace, J. B., Eggert, S. L., Meyer, J. L., & Webster, J. R. (1997). Multiple trophic levels of a forest stream linked to terrestrial litter inputs. *Science,**277*(5322), 102–104. 10.1126/science.277.5322.102

[CR83] Yule, C. M., Boyero, L., & Marchant, R. (2010). Effects of sediment pollution on food webs in a tropical river (Borneo, Indonesia). *Marine and Freshwater Research,**61*, 204–213. 10.1071/MF09065

